# Abscess Size and Depth on Ultrasound and Association with Treatment Failure without Drainage

**DOI:** 10.5811/westjem.2019.12.41921

**Published:** 2020-02-26

**Authors:** Frances M. Russell, Matt Rutz, L. Ken Rood, Justin McGee, Elisa J. Sarmiento

**Affiliations:** Indiana University, Department of Emergency Medicine, Indianapolis, Indiana

## Abstract

**Introduction:**

Skin and soft tissue infections (SSTI) occur along a continuum from cellulitis to abscess. Point-of-care ultrasound (POCUS) is effective in differentiating between these two diagnoses and guiding acute management decisions. Smaller and more superficial abscesses may not require a drainage procedure for cure. The goal of this study was to evaluate the optimal abscess size and depth cut-off for determining when a drainage procedure is necessary.

**Methods:**

We conducted a retrospective study of adult patients with a SSTI who had POCUS performed. Patients were identified through an ultrasound database. We reviewed examinations for the presence, size, and depth of abscess. Medical records were reviewed to determine acute ED management and assess outcomes. The primary outcome evaluated the optimal abscess size and depth when a patient could be safely discharged without a drainage procedure. We defined a treatment failure as a return visit within seven days requiring admission, change in antibiotics, or drainage procedure.

**Results:**

A total of 162 patients had an abscess confirmed on POCUS and were discharged from the ED without a drainage procedure. The optimal cut-off to predict treatment failure by receiver operating curve analysis was 1.3 centimeters (cm) in longest dimension with a sensitivity of 85% and specificity of 37% (area under the curve [AUC] 0.60, 95% confidence interval [CI], 0.44–0.76), and 0.4cm in depth with a sensitivity of 85% and specificity of 68% (AUC 0.83, 95% CI, 0.74–93).

**Conclusion:**

This retrospective data suggests that abscesses greater than 0.4 cm in depth from the skin surface may require a drainage procedure. Those less than 0.4 cm in depth may not require a drainage procedure and may be safely treated with antibiotics alone. Further prospective data is needed to validate these findings and to assess for an optimal size cut-off when a patient with a skin abscess may be discharged without a drainage procedure.

## INTRODUCTION

Emergency department (ED) visits for skin and soft tissue infections (SSTI) have markedly increased over the last decade,^1^,^2^ accounting for more than 4.21 million ED visits in 2010 alone.^3^ SSTIs occur along a continuum from cellulitis to abscess. In patients with suspected SSTI, point-of-care ultrasound (POCUS) is effective in differentiating cellulitis vs abscess, in both adult and pediatric populations.^4^–^8^ This is an important distinction as standard treatment for abscess involves an invasive and often painful drainage procedure,^9^–^12^ while cellulitis is commonly treated with antibiotics alone.^13^,^14^ Smaller and more superficial abscesses may heal without a drainage procedure and with antibiotics alone.

Although soft-tissue POCUS is often incorporated into the clinical evaluation of patients with SSTI, there is limited evidence evaluating the impact of abscess size and depth on acute management. It is possible that smaller and more superficial abscesses may be managed without a drainage procedure. We set out to assess the optimal abscess size and depth cut-off, as visualized on POCUS, for determining when a drainage procedure is necessary.

## METHODS

### Study Design

This was a retrospective study of adult patients with a SSTI who received an emergency physician-performed POCUS examination at two urban, academic EDs with a combined volume of >220,000 patient visits per year. We reviewed all soft tissue studies logged into an ultrasound database, Qpath (Telexy Healthcare, British Columbia, Canada), between September 2013 and July 2019. This study was approved by the institutional review board with waiver of consent.

**Population Health Research Capsule**

What do we already know about this issue?

Point-of-care ultrasound can reliably differentiate cellulitis from abscess in patients with skin and soft tissue infections.

What was the research question?

The primary aim was to evaluate the optimal abscess size and depth cut-off for determining when a drainage procedure is necessary.

What was the major finding of the study?

Skin abscesses >0.4 centimeters (cm) in depth may require a drainage procedure, while those <0.4 cm may be safely treated with antibiotics alone. Additional data is needed to determine an optimal size cut-off for when a drainage procedure is not necessary.

How does this improve population health?

Superficial abscesses (<0.4 cm deep) may be effectively treated without a drainage procedure, obviating the need for a time-consuming and invasive procedure.

We included all adult patients who presented to the ED with signs or symptoms that prompted an emergency physician to perform a soft tissue POCUS. We included patients with a skin abscess, defined as a well-circumscribed fluid collection with posterior acoustic enhancement. We excluded patients without abscess (i.e., cellulitis alone, simple cysts, lymph node, etc.), those with a peritonsillar or breast abscess, patients requiring hospital admission, patients whose demographics were entered incorrectly into the ultrasound database (i.e., we could not identify the patient), and incarcerated patients (Figure).

### Study Protocol

Four emergency physicians, including three ultrasound-trained faculty and one senior resident, reviewed previously performed POCUS examinations for the presence of abscess. Ultrasound images included both video and/or still images. Some images included the measurements of the abscess including height, length, width, and depth. If the images did not include measurements or were measured incorrectly, the reviewers performed their own measurements for size and depth.

If identified, the longest diameter in any dimension and depth from the skin surface to the superficial edge of the abscess were measured and recorded on a standardized data collection form. These same emergency physician reviewers then collected patient demographic information, abscess location, whether the patient was immunocompromised, and whether the patient was using intravenous drugs. Immunocompromised states were defined as patients with diabetes, human immunodeficiency virus, or on immunosuppressant medication. Patients’ statewide electronic health records, a database external to the hospital electronic health record, were reviewed to determine whether an incision and drainage (I&D) procedure had been performed and to assess seven-day outcomes.

Reviewers followed previously published methods for reviewing charts.^15^ This included pre-study training on where to extract data, a standardized data abstraction form, and defining variables pre-study. Study monitoring was performed periodically, after 50 and 100 patients, to ensure all variables were being collected in the same format. Reviewers were not blinded to the study hypothesis. A second investigator reviewed a randomized sample of 64 (15%) patient images to assess for intraclass correlation for abscess size and depth. The second investigator was blinded to prior measurements, patient history and outcome data.

### Outcome

The primary outcome was to determine the impact abscess length and depth had on outcomes of ED patients discharged without a drainage procedure. In those patients with an abscess who did not undergo a drainage procedure, we evaluated the optimal abscess size and depth cut-point at which patients did not have a treatment failure. A treatment failure was defined as an unscheduled healthcare visit within seven days requiring hospital admission, a change in antibiotic, or a drainage procedure.

### Data Analysis

Continuous data is presented as median with interquartile range (IQR). We calculated percent frequency of occurrence, sensitivity and specificity with 95% confidence intervals (CI). Receiver operating characteristic (ROC) curves were used to determine the optimal cutoff value for both abscess size (longest dimension) and depth from skin surface for an abscess effectively treated without a drainage procedure. We completed statistical analysis using SAS, version 9.4 (SAS Institute, Cary, NC).

## RESULTS

Of 999 patients found to have a skin abscess on POCUS after we applied inclusion/exclusion criteria, 162 (16.2%) were discharged from the ED without a drainage procedure (Figure). The median age was 37.6 years (IQR 21, 18–73), and the median duration of symptoms prior to evaluation was three days (IQR 5). The most common abscess locations were the extremities (51%). Twenty-one (10%) patients had diabetes mellitus and 18 (11%) were intravenous drug users (Table 1). The majority of patients were discharged with either clindamycin (31%) or the combination of cephalexin and trimethoprim/sulfamethozaxole (25%). Eighteen (11%) patients were discharged without drainage or antibiotics.

The training level of sonographers included fourth-year medical students, postgraduate year 1–5 emergency medicine (EM)/EM-pediatric residents and board-certified EM faculty. Sonographers did not use a standardized imaging protocol for ultrasound assessment of SSTI; however, they were taught to scan through the area of interest in two planes, orthogonal to each other.

For the 162 patients discharged without a drainage procedure, 13 (8%) had a treatment failure four required admission; eight a change in antibiotics; and seven a drainage procedure during their subsequent encounter (Table 2). No treatment failures went to the operating room. Of these 162, the median length and depth in centimeters (cm) were 1 cm (IQR 0.9, 0.25–4.2) and 0.25 cm (IQR 0.4, 0–2). The optimal cut-off value to predict treatment failure by ROC analysis was 1.3 cm in longest dimension with a sensitivity of 85% and specificity of 37% (area under the curve [AUC] 0.60, 95% CI, 0.44–0.76). The optimal cut-off value for depth was 0.4cm with a sensitivity of 85% and specificity of 68% (AUC 0.83, 95% CI, 0.74–93). One hundred and six (65.4%) patients had an abscess length less than 1.3cm, and 103 (63.5%) had an abscess depth less than 0.4 cm from the skin surface. The length threshold for 100% sensitivity was 0.47 cm with a specificity of 2%. The depth threshold for 100% sensitivity was 0.2 cm with a specificity of 34% (Table 3).

The intraclass correlation between blinded reviewers for abscess size and depth was 0.92.

## DISCUSSION

POCUS is readily available and currently used in the ED to guide acute treatment decisions in patients with SSTI.^2^ Ultrasound gives clinicians the ability to differentiate between cellulitis and abscess, something that physical examination cannot always do.^4^,^5^ Despite this fact, very little is known about how to manage smaller and shallower skin abscesses. Analyzing the size and depth of an abscess may further impact a patient’s management course. In this study we found that abscesses less than 0.4 cm deep to the skin surface may be effectively treated without a drainage procedure. This is important as standard treatment for abscess typically involves an invasive I&D procedure.^11^,^12^ Our data suggests that more superficial abscesses may be safely and effectively treated without a drainage procedure. These findings may allow clinicians to avoid an unnecessary, time consuming, and invasive procedure in these select patients.

This is the first study to date to assess the impact of size and depth of an abscess on acute ED management in patients who have more than cellulitis, but may not have a large enough abscess to require drainage. Recent studies differ from ours in that they primarily focused on management of uncomplicated SSTI with I&D with or without the addition of oral antibiotics.^11^,^12^,^16^ Talan et al^11^ found that in patients with abscesses with a median length of 2.5 cm and depth of 1.5 cm who were treated with oral trimethoprim-sulfamethoxazole in conjunction with an I&D procedure had a higher cure rate when compared to patients who received an I&D procedure and placebo.

Daum et a.^12^ found that in patients with a skin abscess less than or equal to 5cm in diameter treated with oral antibiotics in combination with I&D had improved short-term outcomes compared to those patients treated with I&D alone. A systematic review and meta-analysis by Gottlieb et al,^16^ which included the two previously mentioned studies, found that the addition of antibiotics to a drainage procedure improved clinical cure in patients with a SSTI. In all of these studies all patients with an abscess underwent a drainage procedure.

In this study, we found that the optimal cut-off value to predict treatment failure by ROC analysis was 1.3 cm in longest dimension with an AUC of 0.60 (95% CI, 0.44–0.76). Unfortunately, this data is not able to accurately determine an abscess size cutoff. This is likely a reflection of both a small sample size and the low number of treatment failures recorded in our data. Further investigation is needed to better define an optimal size cut-point when a drainage procedure is not indicated and a patient may be safely discharged.

There were only 13 (8%) treatment failures. Seventy-six percent were located on the extremities and trunk. Two patients were immunocompromised with diabetes, and two patients used intravenous drugs. Twelve (92%) were discharged with antibiotics, the majority receiving either clindamycin or cephalexin with trimethoprim/sulfamethozaxole. One abscess was 1.75 cm deep to the skin surface and another was 3 cm in length, which may account for their failure. One patient had a treatment failure with a depth and length of 0.4 cm. The abscess did not require drainage, but a change in antibiotics and admission. It is possible that in this case the type of infection played a role in treatment failure. A different patient had a treatment failure with a depth of 0.2 cm and a length of 0.4 cm. This abscess was located on the face, and it is possible that in this case the location led to more aggressive treatment when the patient returned.

There are some limitations to POCUS for SSTIs. Ultrasound image acquisition and interpretation rely on the sonographer’s ability to acquire high-quality images to be able to assess whether an abscess is present. An abscess may appear hypoechoic, hyperechoic or even anechoic, and will typically have posterior acoustic enhancement. Additionally, more complicated infections such as necrotizing fasciitis will have subcutaneous thickening, free fascial fluid, and/or subcutaneous air. It is important for the sonographer to be familiar with different findings on soft tissue ultrasound to guide appropriate treatment.

Future research aimed at prospectively assessing which abscesses can safely be treated without a drainage procedure is needed. Future studies should seek to include pre-study training in soft tissue ultrasound with a standardized scanning approach, a larger sample size, consecutive patients, and structured follow-up.

## LIMITATIONS

This study has a number of limitations that may affect its generalizability. It is a retrospective study using a pre-existing database of images. There was potential for selection bias as patients were recruited by convenience sampling and may not have represented the general population. To be included in the study patients had to have a soft tissue ultrasound performed and images saved. These images were acquired by sonographers with varying levels of training. It is possible we missed patients who could have been included in the study as no images were saved or due to incorrect/no patient information. We also excluded a large number of patients who received a drainage procedure, including those with smaller abscesses (<1 cm), as this is the most common treatment for an abscess. It is possible that some of these abscesses did not require a drainage procedure. Future studies should include a pre-defined scanning protocol and treatment algorithm based on ultrasound findings to guide in determining which patients should or should not receive a drainage procedure.

Additionally, the number of patients with a treatment failure was relatively small resulting in large CIs, and poor ROC analysis for longest dimension with an AUC of 0.60. Despite this, the AUC for depth was 0.83 suggesting that a depth of 0.4 cm is a good cut-point to be able to differentiate between patients who will or will not fail treatment without a drainage procedure. It is unclear whether the two measurements, length and depth, had an influence on each other with regard to treatment failure. Lastly, as this was a retrospective study we did not collect any data on the type of bacterial infection or control for the antibiotics prescribed. It is possible there are additional unidentified confounders. The data presented in this study raises further questions that should be explored in future prospective studies.

## CONCLUSION

This small retrospective study suggests that a skin abscess less than 0.4 cm deep to the skin surface may be treated successfully without an invasive drainage procedure. Those deeper than 0.4 cm may require a drainage procedure. Further data is needed to validate these findings and to assess for an optimal size cut-off when a patient with a skin abscess may be discharged without a drainage procedure.

## Figures and Tables

**Figure:**
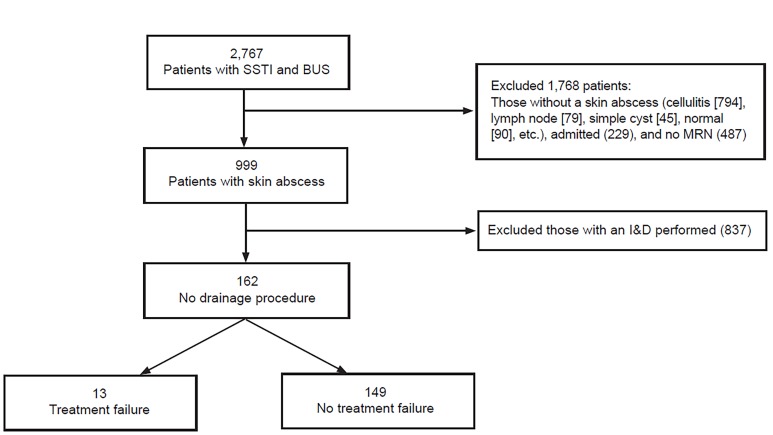
Patient flow, treatment, and outcomes. *SSTI*, skin and soft tissue infection; *BUS*, bedside ultrasound; *MRN*, medical record number; *I&D*, incision and drainage.

**Table 1:** Characteristics of patients without a drainage procedure.

	Total n=162	No treatment failure n=149	Treatment failure n=13
Age (yrs)			
Median (IQR)	37.6 (21)	36 (20)	47 (15)
Range	18–73	18–73	24–64
Race (%)			
White	69 (42.6%)	62 (41.6%)	7 (54%)
Black	66 (41%)	61 (41%)	5 (38.5%)
Hispanic	12 (7.4%)	12 (8%)	0
Other/Unknown	15 (9.3%)	14 (9.4%)	1 (7.7%)
Duration (days of symptoms)			
Median (IQR)	3 (5)	3 (5)	3 (1)
Location			
Extremity	83 (51%)	78 (52%)	5 (38.4%)
Trunk	51 (31%)	46 (31%)	5 (38.4%)
Head/neck	27 (17%)	24 (16%)	3 (23%)
Unknown	1 (1%)	1 (1%)	0
Immunocompromised			
Diabetes	21 (10%)	19 (10%)	2 (15%)
HIV	1 (0.6%)	1 (0.7%)	0
Other	7 (4%)	7 (5%)	0
IVDU	18 (11%)	16 (10.7%)	2 (15%)
Antibiotics at discharge			
Clindamycin	50 (31%)	44 (30%)	6 (46%)
Cephalexin & TMP/Sulfa	41 (25%)	38 (26%)	3 (23%)
TMP/Sulfa	17 (10%)	16 (11%)	1 (8%)
Cephalexin	15 (9%)	15 (10%)	0
Other	21 (13%)	19 (13%)	2 (15%)
None	18 (11%)	17 (11%)	1 (8%)

*HIV*, human immodeficiency virus; *IQR*, interquartile range; *IVDU*, intravenous drug use; *Sulfa*, sulfamethozaxole; *TMP*, trimethoprim.

**Table 2:** Characteristics of patients with a treatment failure.

Patient number	Patient age (yrs), risk factors	Abscess Characteristics	Reason for treatment failure
1	24; none	Duration: 3; Location: Buttock Length:1; Depth:1	Change in antibiotics
2	27; none	Duration: 1; Location: Face Length:0.4; Depth:0.4	Change in antibiotics, Admission
3	53; none	Duration: 2; Location: Face Length:0.61; Depth:1.75	Change in antibiotics
4	35; none	Duration: 3; Location: Arm Length:3; Depth:0.5	I&D
5	46; none	Duration: 3; Location: Labia Length:0.5; Depth:0.7	Admission
6	50; Diabetes	Duration: 3; Location: Scrotum Length:1.2; Depth:0.3	Change in antibiotics, I&D
7	47; none	Duration: 7; Location: Buttock Length:0.7; Depth:0.5	Change in antibiotics, I&D
8	64; Diabetes	Duration: 2; Location: Arm Length:1.2; Depth:1	Change in antibiotics, Admission
9	36; IVDU	Duration: 3; Location: Arm Length:1.1; Depth:0.7	I&D
10	50; IVDU	Duration: 1; Location: Face Length:0.4; Depth:0.2	Change in antibiotics, I&D, Admission
11	48; none	Duration: 7; Location: Leg Length:0.5; Depth:0.5	Change in antibiotics
12	26; none	Duration: 3; Location: Leg Length:1.5; Depth:0.5	I&D
13	52; none	Duration: 1; Location: Buttock Length:1; Depth:1	I&D

Duration is in days; length/depth are in centimeters.

*IQR*, interquartile range; *IVDU*, intravenous drug use; *I&D*, Incision and drainage.

**Table 3:** Sensitivities and specificities at different cutoffs for length and depth.

Length (cm)	0.25	0.5	0.75	1	1.3	1.5
Sensitivity	100%	92%	92%	92%	85%	62%
Specificity	0%	5%	17%	24%	37%	45%
PPV	8%	8%	9%	10%	11%	9%
NPV	100%	88%	96%	97%	96%	93%
Depth (cm)	0	0.2	0.25	0.4	0.5	1
Sensitivity	100%%	100%	92%	85%	77%	31%
Specificity	0%	34%	53%	68%	75%	97%
PPV	8%	12%	15%	19%	21%	44%
NPV	100%	100%	99%	98%	97%	94%

*PPV*, positive predictive value; *NPV*, negative predictive value; *cm*, centimeter.
